# A Comparative Study of Four Types of Free Flaps from the Ipsilateral Extremity for Finger Reconstruction

**DOI:** 10.1371/journal.pone.0104014

**Published:** 2014-08-06

**Authors:** Yujie Liu, Hongsheng Jiao, Xiang Ji, Chunlei Liu, Xiaopen Zhong, Hongxun Zhang, Xiaohen Ding, Xuecheng Cao

**Affiliations:** 1 Department of Orthopedic and Traumatic Surgery, General Hospital of Jinan Military Command, Jinan, P. R. China; 2 The Hand Surgery Center of Chinese People’s Liberation Army, The 401^st^ Hospital of CPLA, Qingdao, P. R. China; di Pompeo d’Illasi, Sapienza, University of Rome, School of Medicine and Psychology, Italy

## Abstract

**Aim:**

To compare the outcomes of finger reconstruction using arterialized venous flap (AVF), superficial palmar branch of the radial artery (SPBRA) flap, posterior interosseous perforator flap (PIPF), and ulnar artery perforator free (UAPF) flap harvested from the ipsilateral extremity.

**Methods:**

We retrospectively reviewed the outcomes for 41 free flaps from the ipsilateral extremity in the reconstruction of finger defects in 41 patients with small/moderate skin defects, including 11 AVFs, 10 SPBRA flaps, 10 PIPFs, and 10 UAPF flaps. Standardized assessment of outcomes was performed, including duration of operation, objective sensory recovery, cold intolerance, time of returning to work, active total range of motion (ROM) of the injured fingers, and the cosmetic appearance of the donor/recipient sites.

**Results:**

All flaps survived completely, and the follow-up duration was 13.5 months. The mean duration of the complete surgical procedure for AVFs was distinctly shorter than that of the other flaps (*p*<0.05). AVFs were employed to reconstruct skin defects and extensor tendon defects using a vascularized palmaris longus graft in 4 fingers. Digital blood supply was reestablished in 4 fingers by flow-through technique when using AVFs. Optimal sensory recovery was better with AVFs and SPBRA flaps as compared with UAPF flaps and PIPFs (*p*<0.05). No significant differences were noted in ROM or cold intolerance between the 4 groups. Optimal cosmetic satisfaction was noted for the recipient sites of AVFs and the donor sites of SPBRA flaps. The number of second-stage defatting operations required for AVFs was considerably lesser than that for the other flaps.

**Conclusion:**

All 4 types of free flaps from the ipsilateral extremity are a practical choice in finger reconstruction for small/moderate-sized skin defects. AVFs play an important role in such operations due to the wider indications, and better sensory recovery and cosmetic appearance associated with this method.

## Introduction

The reconstruction of fingers with skin and soft tissue defects remains challenging. The optimal reconstructive treatment should be simple, reliable, cost effective, and provide pliable, sensitive, and cosmetically similar tissue that will allow adequate function [1]. Local and regional skin flaps, such as the palmar advancement flap [2], cross-finger flap [3], distally based homodigital island flaps [4], and pedicled perforator finger flaps [5], [6], are excellent for rapid and easy reconstruction; however, they do involve certain drawbacks, such as limitations of flap advancement or coverage, poor sensation joint stiffness, large scars on donor and recipient digits, vulnerable venous return, and the potential for development of painful neuroma in the pedicle [3]–[6].

Although the concept of free tissue transfer to traumatized digits remains unpopular with many surgeons for high technique demand, a free flap of appropriate size may provide an ideal surgical solution, since it is associated with a shorter time of returning to work and satisfactory function and aesthetic appearance [7]. According to the “replace like with like” principle [8], free flaps used for the repair of the finger skin defects should ideally be obtained from the counterparts of the fingers–i.e., the toes. However, the markedly high rate of donor site morbidity is the main disadvantage of the free pulp flap [7], [9], [10]. Several types of distant free flaps are available for reconstructing finger injuries, such as the posterior auricular free flap [11] and the medial plantar artery perforator flap [12]. However, these procedures require two operative fields and complex anesthesia.

Four types of free flaps have been used in finger reconstruction from the ipsilateral extremity, including arterialized venous flap (AVF) [13], superficial palmar branch of the radial artery (SPBRA) flap [14], posterior interosseous perforator flap (PIPF) [15], and ulnar artery perforator free (UAPF) flap [16]. These flaps are characterized by the following features: (i) they need only one operative field and simple anesthesia involving a simple brachial block to the injured extremity, which is surgeon-friendly with a single tourniquet; (ii) low donor site morbidity, and without sacrificing the main vessels; (iii) allowing sensory recovery in the fingers due to the inclusion of a sensory nerve; and (iv) thin flap which can achieve better aesthetic appearance due to less subcutaneous fat [13]–[16].

However, in clinical practice, the selection of these flaps remains contentious since no study has investigated the differences in the clinical outcomes of these 4 free flaps. In this retrospective study, we compared and analyzed the outcomes in 41 patients with small/moderate skin defects who underwent finger reconstruction using AVF, SPBRA flap, PIPF, or UAPF flap.

## Patients and methods

### Patients

We included 41 patients admitted to our department from October 2006 to December 2012 in this retrospective analysis. Among these cases, finger reconstruction using AVFs was performed in 11 patients, while SPBRA flaps, PIPFs, or UAPF flaps were used for single finger injury in 10 patients. All the patients had sustained skin defects with the exposure of the deep structures, such as tendons, bones, or joints. All the patients were treated as emergencies within an average of 6 hours after injury (range, 2–10 h). The mechanisms of injury included crushing injury, degloving injury, and cutting injury (Table 1). Innervated flaps were employed in reconstruction only when all the finger pulps were damaged. Among the 41 fingers, 2 fingers for each flap type (n = 8) with finger pulp defects were constructed by a sensate flap. In other cases, nonsensate flaps were used.

**Table 1:** Demographic data.

Group	Flaps (n)	Age	Sex	Sex	Injurymechanism			Finger		Extensortendondefect	bilateral arteriadigitalis defect
			Male	Female	CU	CR	DE	DF	Non-DF		
AVF group	11	31±7.2 (17–44)	7	4	4	4	3	6	5	4	4
SPBRA flap group	10	34±8.9 (19–42)	7	3	2	6	2	6	4	none	none
UAPF group	10	36±6.7 (16–45)	7	3	1	7	2	6	4	none	none
PIPF group	10	35±6.2 (17–42)	6	4	2	6	2	5	5	none	none

AVF: arterialized venous flap, SPBRA: superficial palmar branch of the radial artery flap, PIPF: posterior interosseous perforator flap, UAPF: ulnar artery perforator free flap, CR: crushing injury, DE: degloving injury, CU: cutting injury, DF, fingers of the dominant hand, non-DF, fingers of the non-dominant hand.

Written informed consent was obtained from each patient prior to surgery and saved in the documentation department. The protocols used in this study were approved by the Ethics Committee of the 401^st^ Hospital of Chinese People Liberation Army (Qingdao, China).

### Methods

Cases were reviewed in terms of objective sensory recovery, cold intolerance, and time of returning to work. Sensory testing was undertaken using static two-point discrimination (s2PD), moving two-point discrimination (m2PD), and Semmes-Weinstein monofilament test (SWM test). The overall outcomes of the patients were assessed independently by the senior author (J.X.), who was blinded to the surgical procedure. Cold intolerance in the reconstructed digit was rated by the patients based on normal daily activity and graded as none, slight, moderate, or severe [17]. Patients’ self-assessments for cosmetic appearance–mainly based on the appearance of the donor and recipient sites were carried out with a visual analog scale ranging from 0 (completely disappointed) to 10 (completely satisfied) and divided into 3 classes (good, 10–8; acceptable, 7–5; unacceptable, <5) [18], [19].

### Statistical analysis

The F-test was used to assess the homogeneity of variance of the demographic data for the 2 groups. Student’s *t*-test was used to compare intergroup differences in the duration of operation and time of returning to work. The Wilcoxon signed-rank test was used to compare intergroup differences in cold intolerance, 2PD, and SWM tests. A *p* value <0.05 was considered to demonstrate statistically significant differences.

### Surgical techniques

All the flaps were harvested from the forearm of the ipsilateral extremity under a brachial plexus nerve block. The surgery was performed using a pneumatic tourniquet. After thorough debridement, the recipient digital artery and nerve and the dorsal digital vein were identified and marked. All the flaps were tailored to a size 5–8 mm larger than the recipient site to alleviate possible postoperative swelling and edema.

#### Designing and harvesting the AVF

AVF elevation was performed as previously described [13]. Briefly, the flap was designed on the volar side of the forearm, which included 2 veins. The ratio of the afferent and efferent veins to be anastomosed was 1∶1 or 1∶2. The relatively smaller vein was used as the afferent vein, while the larger vein was used as the efferent vein. In total, 11 AVFs were harvested, among which 7 were allocated to type III (perfusion patterns: A-V-V, 1 through-valve and 6 against-valve), and 4 were allocated to type IV (perfusion patterns: A-V-A, 4 through-valve) according to Chen’s classification [20]. The efferent vein was delivered to the dorsal anastomotic site through a loose subcutaneous tunnel. In 4 fingers, AVFs combined with a vascularized palmaris longus graft were employed to reconstruct extensor tendon defects simultaneously. In 4 other fingers, the digital blood supply was lost since the bilateral arteria digitalis vessels were severed. Digital blood supply was reestablished via the flow-through technique using the veins contained in AVF grafts. The anterior branch of the medial or lateral cutaneous nerve of the forearm was incorporated into the flap to create an innervated flap if necessary.

#### Designing and harvesting the SPBRA flap

The SPBRA flap was elevated as previously described [14]. Briefly, after the route of the flap and subcutaneous vein were marked, the SPBRA flap was designed over the volar aspect of the distal forearm according to the size of the finger defect. The flap was designed with an elliptical shape to facilitate donor site closure. The concomitant vein of the SPBRA flap and subcutaneous veins were preserved to facilitate subsequent venous return. The palmar cutaneous branch of the median nerve was incorporated into the flap to create an innervated flap if necessary.

#### Designing and harvesting the UAPF flap

The UAPF flap was raised as previously described [16], [21]. In brief, the axis of the UAPF flap was the connecting line between the pisiform and the medial humeral epicondyle. The ulnar artery perforator arose from a branch of the ulnar artery, located approximately 40 mm proximal to the pisiform bone. The flap was designed according to the size of the defect. Then, an incision was made along the radial border of the flap. Once the perforator was identified, an incision was made for the ulnar border of the flap, and the flap was elevated. The accompanying vein and superficial vein in the flap were used for venous return. The terminal branch of the medial antebrachial cutaneous nerve was incorporated into the flap to create an innervated flap if necessary.

#### Designing and harvesting the PIPFs

PIPFs were designed as described previously after the perforator was preoperatively identified by Doppler examination [15]. The PIPF was elevated from the ulnar side to the radial side through the subcutaneous tissue plane. Once the perforator was identified, the intermuscular septum and a tiny ellipse of the deep fascial cuff were preserved around the posterior interosseous vessel. In some patients, the diameter of the accompanying vein was too narrow; therefore, the superficial vein was instead used for ensuring venous return. The posterior antebrachial cutaneous nerve was incorporated into the flap to create an innervated flap if necessary.

Upon the completion of flap grafting in the recipient sites, anastomoses of the blood vessels and/or nerves were performed using an end-to-end method. Postoperatively, each flap was monitored hourly for 3 days. Each patient received oral aspirin (125 mg/day) and subcutaneously injected low-molecular-weight dextran (30 mL/day) for 7 days postoperatively. Further, a dorsal cast of plaster of Paris was used for immobilizing the injured limb for 1 week. Subsequently, an active and passive physical rehabilitation program was initiated to achieve the finger’s maximal range of motion (ROM).

## Results

All the patients included in the study were followed up for a mean duration of 13.5 months (9–18 months). The outcome was recorded in each group and the details of complications have been provided in Table 2. The mean sizes of the AVF, SPBRA flap, UAPF flap, and PIPF were 35×19 mm, 34×16 mm, 31×19 mm, and 34×20 mm, respectively. All the flaps survived completely. All donor sites were closed primarily without dehiscence except for 4 flaps, including 2 UAPF flaps (measuring 5×3 cm and 5×3.5 cm) and 2 SPBRA flaps (measuring 5×3.2 cm and 4×2.6 cm). Full-thickness skin grafts were used to close these donor sites. The mean duration of the complete surgical procedure of AVF was 3.4±1.2 (3.0–4.5) h, which was distinctly shorter than that for the other flaps (*p*<0.05).

**Table 2:** General results.

Group	Flaps(n)	Flapsize (mm)	Flapsurvival	Meansurgicalduration(hour)	Time ofreturningto work(week)	Coldintolerance				Patients’self-assessmentsfor cosmeticappearance		Mean ofROM (°)	Number offlaps neededfor secondarysurgery fordefatting
						None	Slight	Moderate	Severe	Recipient Site	Donor Site		
AVFgroup	11	35×19	CS, 6 blisterformation	3.4±1.2 (3.0–4.5)	12 (11–21)	2	5	2	2	good (9);acceptable (2)	good (4);acceptable (7)	208	none
SPBRAflap group	10	34×16	CS, 3 blisterformation	4.9±1.7 * (4.1–6.5)	10 (7–15)	7	1	2	1	good (5);acceptable (5)	good (8);acceptable (2)	233	4
UAPFgroup	10	31×19	CS, 2 blisterformation	5.1±1.3 * (4.5–6.1)	9 (6–15)	6	2	2	0	good (3);acceptable (7)	good (5);acceptable (5)	224	5
PIPFgroup	10	34×20	CS, 3 blisterformation	4.8±1.8 * (4.0–6.7)	10 (7–16)	6	1	3	0	good (3);acceptable (7)	good (5);acceptable (5)	214	5

*, P<0.05, compared with the AVF group; AVF: arterialized venous flap, SPBRA: superficial palmar branch of the radial artery flap, PIPF: posterior interosseous perforator flap, UAPF: ulnar artery perforator free flap, CS: complete survival, ROM: range of motion. Surgical duration: from the induction of anesthesia until the patient was transferred from the operating room.

AVF grafts were more prone to blister formation as compared to the other graft types. In the AVF grafts, blister formation was observed in 6 flaps postoperatively (6/11), but only in 2–3 flaps from each of the other groups. All the blisters subsided gradually with no special care. In the AVF grafts, blisters were formed in a retrograde perfusion pattern in 1 flap, while in the other 5 flaps, they were formed in an antegrade perfusion pattern.

Almost full ROM was obtained in all the reconstructed fingers. The mean ROM was 198° in the 4 fingers where the vascularized palmaris longus tendon graft was used with AVF. Two fingers using AVFs showed moderate cold intolerance, while the 2 patients in whom the bilateral arteria digitalis were damaged demonstrated severe intolerance. No significant differences were noted in ROM or cold intolerance between the 4 groups (*p*>0.05).

The results of the sensory evaluation of the 4 types of nonsensate flaps are shown in Table 3. In the 33 grafts with nonsensate flap, good sensory recovery was obtained in the patients who received AVF and SPBRA flaps, with s2PD of 7 mm (4–9 mm) and 8 mm (6–9 mm), respectively (Table 3). However, poor sensation was recorded for the fingers reconstructed using UAPF flaps and PIPF, with s2PD of 11 mm (7–14 mm) and 13 mm (8–16 mm), respectively. With regard to SWM, a higher percentage of normal sensation (filament level, 2.36–2.83) was noted in the grafts with AVF (3/9, 33.3%) and SPBRA flap (2/8, 25%). In contrast, no normal sensation was noted in UAPF flap and PIPF. Diminished light touch was achieved only in 1 flap each in the grafts with UAPF flap (1/8, 12.5%) and PIPF (1/8, 12.5%). For intergroup differences for s2PD, m2DP, and SWM, the Wilcoxon signed-rank test demonstrated significant differences in sensory recovery for each parameter (*p*<0.05). The fingers with AVF grafts showed optimal sensory recovery, followed by those with SPBRA flaps. Eight finger pulps (2 from each group) reconstructed by using sensate flaps showed s2PD of 5 mm (5–8 mm), normal sensation (filament level, 2.36–2.83) for 4 fingers, and diminished light touch (filament level, 3.22–3.61) for 4 fingers (Table 4).

**Table 3:** Sensory evaluation results of 4 types of nonsensate flaps.

Group	Flaps (n)	s2PD	m2PD	SWM			
				NS (n)	DLT (n)	DPS (n)	LPS (n)
AVF group	9	7 (4–9)	6 (4–8)	3	3	2	1
SPBRAflap group	8	8 (6–9)	7 (5–9)	2	3	2	1
UAPF group	8	11 (7–14)* ^,^ #	12 (6–13) * ^,^ #	0	1	4	3
PIPF group	8	13 (8–16)* ^,^ # ^,^ †	11 (8–15) * ^,^ #	0	1	3	4

*, P<0.05, compared with the AVF group;

# , P<0.05, compared with the SPBRA group;

† , and P<0.05, compared with the UAPF group.

AVF: arterialized venous flap, SPBRA: superficial palmar branch of the radial artery, PIPF: posterior interosseous perforator flap, UAPF: ulnar artery perforator free flap, SWM: Semmes-Weinstein monofilament test, NS: normal sensation (filament level, 2.36–2.83), DLT: diminished light touch (filament level, 3.22–3.61), DPS: diminished protective touch (filament level, 3.84–4.31), LPS: loss of protective sensation (filament level, 4.56).

**Table 4:** Sensory evaluation results of 4 types of sensate flaps.

Group	Flaps (n)	s2PD	m2PD	SWM			
				NS (n)	DLT (n)	DPS (n)	LPS (n)
AVF group	2	5 5	5 4	1	1		
SPBRAflap group	2	6 5	6 5	1	1		
UAPF group	2	6 7	6 6	1	1		
PIPF group	2	7 8	6 7	1	1		

AVF: arterialized venous flap, SPBRA: superficial palmar branch of the radial artery, PIPF: posterior interosseous perforator flap, UAPF: ulnar artery perforator free flap, SWM: Semmes-Weinstein monofilament test, NS: normal sensation (filament level, 2.36–2.83), DLT: diminished light touch (filament level, 3.22–3.61), DPS: diminished protective touch (filament level, 3.84–4.31), LPS: loss of protective sensation (filament level, 4.56).

**Figure 1:**
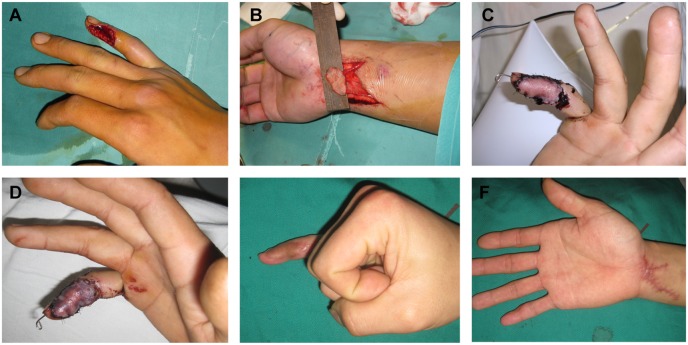
Finger reconstruction by AVF. Case 1: A 34-year-old man underwent finger reconstruction by AVF. (A) Preoperative defect of the little finger. (B) The design and elevation of the AVF. This flap contained 2 veins. The relatively smaller vein was used as the afferent vein, while the larger vein was used as the efferent vein. The perfusion pattern employed was the against-valve type. (C) The 4-day postoperative view shows good blood supply in the flap. (D) The 7-day postoperative view indicates the presence of blisters sporadically distributed over the flap, along with slight venous congestion. (E, F) The 10-month postoperative view shows that all the blisters subsided gradually without any special care. The flap completely survived, with excellent contour and texture. The patient’s self-assessments for cosmetic appearance was good on recipient site (9 scores), acceptable on donor site (6 scores).

**Figure 2:**
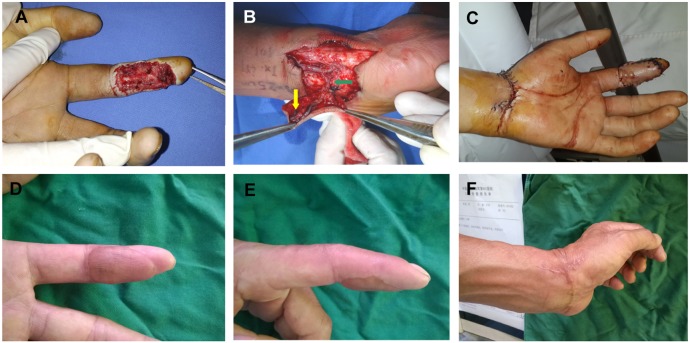
Finger reconstruction by using the SPBRA flap. Case 2: A 45-year-old man underwent finger reconstruction using the SPBRA flap. (A) Preoperative defect of the middle finger. (B) The elevation of the SPBRA flap. The green arrow indicates the SPBRA and its concomitant vein. The yellow arrow indicates a subcutaneous vein. The ratio of the artery and veins to be anastomosed was 1∶2. (C) The 5-day postoperative view indicates the presence of blisters distributed over the flap. All the blisters subsided gradually without any special care. (D) The 10-month postoperative volar view. (E) The 10-month postoperative lateral view. (F) The 10-month postoperative donor site and wrist function view. The patient’s self-assessments for cosmetic appearance was acceptable on recipient site (7 scores), good on donor site (9 scores).

**Figure 3:**
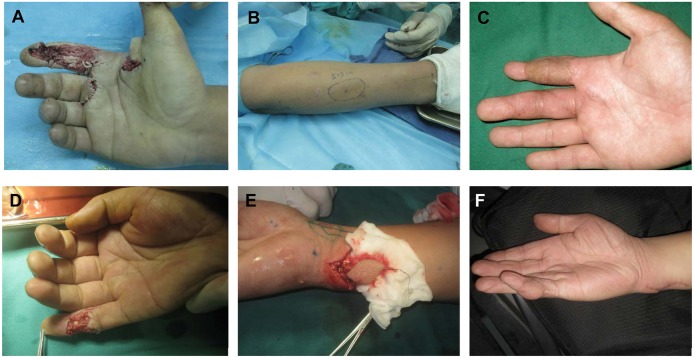
Finger reconstruction by using the PIPF and UAPF flap. Case 3: A 30-year-old man underwent finger reconstruction using the PIPF. (A) Preoperative defect of the index finger. The ulnaris digital artery was intact, whereas a defect of the radialis digital artery was noted. (B) The design of the flap, showing the perforator located at midpoint of Lister's tubercle and humerus epicondyle. The radialis digital artery was anastomosed with the posterior interosseous perforator. The diameter of the accompanying vein was too narrow; therefore, 2 superficial veins were used for ensuring venous return. (C) The 12-month postoperative view. This patient’s self-assessments for cosmetic appearance was good on recipient site (8 scores). Case 4: A 42-year-old man underwent finger reconstruction using the UAPF flap. (D) Preoperative defect of the little finger. (E) The design and elevation of the flap, with the defected ulnar digital artery anastomosed with the ulnar artery perforator, which was located approximately 40 mm proximal to the pisiform bone. The accompanying vein and superficial vein were used for venous return. (F) The 15-month postoperative view. This patient’s self-assessments for cosmetic appearance were acceptable on recipient site (6 scores).

At 9–18 months postoperatively, the patients self-evaluated cosmetic recovery: for the recipient site, the AVF grafts reported the highest satisfaction (9/11, 81.8%), while for the donor sites, the SPBRA flaps were rated the highest (8/10, 80.0%, Table 2). No defatting operations were required in the fingers grafted with AVFs; however, the number of flaps that needed secondary defatting when fingers were grafted with the SPBRA flap, UAPF flap, and PIPF was 4, 5, and 5, respectively (Table 2, Figures 1, 2, 3).

## Discussion

In our study, we found that AVF could be used for broader therapeutic indications than the other 3 flaps. First, composite AVF with a vascularized tendon was an optimal choice for one-stage reconstruction of dorsal composite finger injuries as compared with multi-stage reconstruction or grafting of non-vascularized tendons [22], [23]. Recent studies have indicated that a vascularized tendon can be integrated into AVFs and SPBRA flaps for finger reconstruction of skin and tendon defects [24], [25]. In theory, a vascularized palmaris longus in the SPBRA flap is possible; however, its clinical application remains a problem due to the long distance (average, 2.2–3.0 cm) between the originating point of the SPBRA and the palmaris longus [24]. For a flap with a vascularized palmaris longus tendon, the length of the SPBRA flap must be greater than 3 cm. This limits the clinical application of this flap [14]. In contrast, AVF can be designed in a position centered on the palmaris longus tendon. It is easy to carry the vascularized tendon. In our study, AVFs were used in 4 fingers with extensor tendon defects, with satisfactory outcomes.

Second, AVFs could conveniently cover the wound surface, and it was possible to repair arterial defects via the flow-through technique. According to previous studies, AVFs and SPBRA flaps can be used as flow-through type flaps to reconstruct arterial defects along with skin defects, without sacrificing the main vessels. Iwuagwu et al reported that the SPBRA flap can be used as a flow-through flap to reconstruct digital arteries [25]. However, in our study, we found that the length of the SPBRA was not adequate and could not be adjusted for the vascular defects at the recipient sites. Additionally, the distal diameter of the SPBRA was not comparable to that of the recipient vessel when the injured artery was located in Verdan’s injury zone III, IV, or V [26], which could hamper anastomosis. In contrast, the design and harvesting of AVFs was comparatively easier after taking the area of the wound, the length of the vascular defect, and the diameter of the proximal/distal artery into consideration. In our study, 4 fingers underwent flow-through type of AVFs to reconstruct the digit’s blood supply, with satisfactory outcomes.

In our study, fingers receiving AVF grafts were more prone to developing blisters and venous congestion as compared with those receiving grafts with SPBRA flaps, PIPFs, and UAPF flaps. This is primarily because in AVFs, the primary blood supply enters and exits the flap through the venous system. In the AVF-treated fingers, blister formation occurred on 6 fingers, with significant bullae in the fingers (5/5) grafted with a flap in an antegrade perfusion fashion as compared with the finger (1/6) grafted with a flap in a retrograde perfusion fashion. These results were consistent with a previous study by Woo et al [27], [28], who suggested that retrograde perfusion would enhance flap perfusion by enhancing blood flow in the periphery of AVFs, resulting in satisfactory flap survival. If the blood flows through the flap in the original anatomic direction, no resistance is posed by the venous valves. Consequently, most of the blood flows through the central vein in the flap only, which may lead to insufficient perfusion in the peripheral areas of the flap, eventually leading to blister formation or partial necrosis.

In our series, only 8 fingers received sensate flap reconstruction for finger pulp reconstruction. This is because of the following reasons: (i) In theory, all the 4 types of flaps used in this study could carry sensory nerves; however, the nerves contained in the flaps were rather small, being terminal branches of cutaneous nerves. Therefore, the identification and carrying over of the sensory nerves in the flaps during surgery was challenging. (ii) The sensation in the finger pulp is more important than that in other parts of the finger. When reconstruct finger defect apart from finger pulp by sensate free flap, one digital nerve is often sacrificed to perform an end-to-end nerve anastomosis. This can affect the sensation in the finger pulp [7], [11]. Meanwhile, although the end-to-side method of nerve anastomosis can preserve the digital nerve, the achieved sensation in the flap is not always satisfactory [29], [30]. (iii) According to a previous study, the fingers that received nonsensate flaps showed acceptable s2PD, even with no nerve coaptation. In addition, adequate protective sensation was obtained in the fingers [30], [31]. Satisfactory sensory recovery can be obtained using non-innervated flaps for covering finger defects, especially in younger patients, with sensory recovery mainly depending on the following aspects. First, the ingrowth of the nerve ending from the peripheral and the wound bed could provide good sensory recovery when the flap is thin and narrow. Second, the finger is a highly innervated area and contains numerous nerve endings for regeneration. Third, relatively young patients show improved regeneration and recovery [31].

In the present study, among the for 4 types of nonsensate flaps, the fingers receiving grafts with AVFs showed superior sensory recovery as compared with the other types. Yan et al. reported that sensate AVFs resulted in s2PD of 6–13 mm [23]. Woo et al. [32] demonstrated that in 8 cases (8/20) that underwent reconstruction of palmar soft tissue defects using sensate venous flaps, the average s2PD was 10 mm. Additionally, in patients undergoing reconstruction of the dorsum of the hand, an average s2PD of 13 mm or protective sensation was attained [31]. In this study, the averaged s2PD for fingers with AVF grafts was 7 mm, and normal sensation was achieved in 5 of 11 fingers postoperatively. These results demonstrate that AVFs are effective for sensory recovery through nerve regeneration surrounding the recipient site. Yokoyama et al. reported that sensory improvement can be obtained by finger palmar surface reconstruction without grafting of the subcutaneous nerve. He suggested the presence of the reinnervation effect in venous flaps without neurorrhaphy, when reconstructing fingertip defects [33]. Most importantly, venous flaps are thinner than conventional arterial flaps because they consist only of skin, the venous plexus, and subcutaneous fat, which may theoretically facilitate good sensory recovery through nerve regeneration surrounding the recipient site [34].

Interestingly, satisfactory sensory recovery was also obtained in the SPBRA flap group. Sensory recovery is better when the number of sensory nerves contained in the flap is greater [35], [36]. We speculated that this result was associated with the abundant number of sensory nerves contained in these flaps, i.e., the palmar cutaneous branch of the median nerve, the branches of the superficial radial nerve, and/or the lateral antebrachial cutaneous nerve can be included in SPBRA flaps [24]. In contrast, the sensory recovery in the fingers receiving grafts of UAPF flaps and PIPFs was poor, mainly due to the few sensory nerves contained in those flaps. Based on these results, we propose that AVFs and SPBRA flaps integrated with sensory nerves should be used for the reconstruction of skin defects on the palmar side of the fingers and fingertips.

The shortest mean surgical duration was noted in cases receiving AVFs, including 4 fingers with an extensor tendon defect and 4 fingers with a bilateral arteria digitalis defect. The AVF design is convenient, and its use obviates the need to identify the vessel by preoperative color Doppler. The elevation of AVFs can be performed quicker than the other type of flaps, as elaborate dissection and careful skin perforator protection is not needed. For the other three types of flaps, it requires a considerable amount of time to identify the perforator and dissect it intraoperatively. This may be the main reason why operations involving the other three types of flaps take considerably longer.

Regarding the cosmetic appearance of the donor/recipient sites, optimal appearance was noted in the AVFs, mainly due to the following reasons: The AVFs were thinner than the SPBRA flaps, PIPFs, and UAPF flaps, which contained skin, subcutaneous tissues, deep fascia, and additional tissues to protect the vascular pedicle, in contrast to only skin and subcutaneous tissue in the AVFs. Further, 5 of the 10 SPBRA flap grafts were graded as good (5/10) for cosmetic appearance of the fingers, which was superior to that for grafting with UAPF flaps and PIPFs, possibly because subcutaneous fat distribution in the wrist and radial sides was thinner than that in the ulnar side of the wrist and the dorsal part of the forearm. Further, the SPBRA flap was designed to be parallel to the wrist’s transverse striations, and only a transverse scar remained after the donor sites were sutured. These factors contributed to the optimal degree of satisfaction with regard to the cosmetic appearance of the donor sites for the SPBRA flaps.

The disadvantage of the 4 free flaps described here includes the technical demands of microsurgery, which are not an obstacle for most hand surgeons. However, compared to the use of traditional pedicled flaps, the long operation duration is a drawback of free flap transfer. Vascular anastomosis is main reason for the longer surgical duration, particularly in cases with complications such as vasospasm. Moreover, venous congestion due to a low amount of venous return is the most common reason for the failure of free flap transfer. In the present study, we attempted to anastomose 2 veins instead of 1 vein to avoid venous congestion. This may be a reason for the longer operative time. However, improvement in the microsurgery skill of the surgeon may reduce the operative time. Nevertheless, the risks caused by longer operative time and donor site dehiscence should be considered; however, these were not observed in our series.

In conclusion, the forearm of the ipsilateral extremity is an acceptable donor site for AVFs, SPBRA flaps, UAPF flaps, and PIPFs for the reconstruction of small- and moderate-sized soft tissue skin defects in the fingers. These flaps are suitable for covering finger defects because they are thin, pliable, and hairless, with low donor site morbidity. The optimal cosmetic appearance was observed for recipient sites in patients with AVF grafts. The vascularized palmaris longus tendon could be incorporated into the flap for reconstructing tendon defects and restoring digital circulation via a flow-through flap. AVFs were most useful among the 4 types of flaps studied here because of the simpler technique, wider range of indications, and better sensory recovery and cosmetic appearance. Among SPBRA flaps, UAPF flaps, and PIPFs, optimal sensory recovery was obtained with SPBRA flap grafts together with satisfactory cosmetic appearance and minimal donor site injury.
